# Synthetic Gene Expression Circuits for Designing Precision Tools in Oncology

**DOI:** 10.3389/fcell.2017.00077

**Published:** 2017-08-28

**Authors:** Angela Re

**Affiliations:** Centre for Sustainable Future Technologies, Istituto Italiano di Tecnologia Torino, Italy

**Keywords:** synthetic circuit, biological engineering, synthetic biology, precision medicine, tumor diagnosis, tumor therapy, drug delivery, drug discovery

## Abstract

Precision medicine in oncology needs to enhance its capabilities to match diagnostic and therapeutic technologies to individual patients. Synthetic biology streamlines the design and construction of functionalized devices through standardization and rational engineering of basic biological elements decoupled from their natural context. Remarkable improvements have opened the prospects for the availability of synthetic devices of enhanced mechanism clarity, robustness, sensitivity, as well as scalability and portability, which might bring new capabilities in precision cancer medicine implementations. In this review, we begin by presenting a brief overview of some of the major advances in the engineering of synthetic genetic circuits aimed to the control of gene expression and operating at the transcriptional, post-transcriptional/translational, and post-translational levels. We then focus on engineering synthetic circuits as an enabling methodology for the successful establishment of precision technologies in oncology. We describe significant advancements in our capabilities to tailor synthetic genetic circuits to specific applications in tumor diagnosis, tumor cell- and gene-based therapy, and drug delivery.

## Introduction

Synthetic biology builds on the transformative assertion that engineering approaches could be used to elucidate design principles of cellular systems and to implement synthetic digital and analog subsystems for a variety of end settings including health applications (Lienert et al., 2014). Since its beginning as a formalized engineering paradigm, which could be envisioned near the turn of the century when bacterial cells were programmed with basic genetic circuits (Gardner et al., 2000; Cameron et al., 2014), synthetic biology has provided a rigorous mechanistic foundation extremely helpful to quantitatively characterize the basic functions that are performed by the simple parts of a system and that collectively dictate the emergence of natural and human-defined phenotypes (Mukherji and van Oudenaarden, 2009; Elowitz and Lim, 2010). Nowadays, synthetic biology has greatly expanded in outlook, arising expectations, and stream of thought owing to the increasing intensive convergence of multifaceted engineering, life science, and biotechnology subfields.

The rapid progresses ensued from basic and applied synthetic biology research hold great promise in many contexts of substantial scientific and economic interest. The objective of this review is to reflect on the applications relevant to develop solutions to some of the challenges put forward by precision oncology. The precision paradigm that is being variously adopted by oncology refers both to the chances for enhanced resolution and clarity in tumor identification as well as to the implementation of therapeutic interventions that could be set up on individual case basis (Jain, 2013; Kis et al., 2015). In this text, we provide an overview of synthetic genetic circuits engineering that apply to precision oncology and take advantage of the tight molecular control operating at multiple levels of gene expression (Vazquez-Anderson and Contreras, 2013; Fern and Schulman, 2017), through signal amplification, feedback, oscillatory, and logic capabilities (Wang et al., 2013; Lienert et al., 2014). Specifically, we show that engineered gene regulatory circuits are widening the assays available to report on tumor state and anti-tumor drug responses as well as to devise localized therapeutic options; for instance, increasingly advanced studies are being published on engineering cell classifiers (Morel et al., 2016; Mohammadi et al., 2017) and synthetic constructs for local payload delivery (Wagner et al., 2016). Furthermore, multiple gene-and cell-based therapy choices enhanced by synthetic biology applications are here described (Lim and June, 2017).

A great deal of efforts has been applied to investigate the rules of gene expression by precise measurements afforded by artificially constructed systems (Mukherji and van Oudenaarden, 2009). Much of the early contributions have focused on detailed and quantitative views of transcriptional regulation (Hockenberry and Jewett, 2012), and proceeded in tandem with experimental breakthroughs such as the use of combinatorial promoter libraries (Gertz et al., 2009). Nevertheless, substantial progress has also been achieved in ascertaining other regulatory mechanisms including post-transcriptional, translational, and post-translational modifications (Isaacs et al., 2004; Grilly et al., 2007). Almost all of these regulatory mechanisms are applicable to design gene regulatory platforms with controllable and predictable behaviors. Building on natural examples of regulatory circuits known to tune transcriptional and post-transcriptional activity (Cora et al., 2017), synthetic devices have demonstrated to modulate malignant phenotypes. Interesting examples here include synthetically engineered microRNAs targeting the MYC proto-oncogene (c-Myc) gene, which were shown to inhibit proliferation and induce apoptosis in bladder cancer cells (Fu et al., 2015), and the usage of aptamers to induce tumor cell death by destabilizing the apoptosis regulator bcl-2 (Soundararajan et al., 2008).

While the approaches to design the synthetic biological circuits that will be described could greatly vary, it is clear that abstraction, standardization (Galdzicki et al., 2014), and modularity (Endy, 2005) have been essential to formalize the design of such a broad range of gene expression systems and to handle biological complexity. Such principles lie behind many synthetic circuits to develop diagnostic and therapeutic tools, where basic parts such as promoters, gene coding sequences, terminators, and ribosome binding sites are assembled into modules such as toggle switches (Gardner et al., 2000; Niederholtmeyer et al., 2013) oscillators, and cascades (Davidsohn et al., 2015) to create predictable and continuously more sophisticated functionalities. The achievement of general and scalable systems (Weinberg et al., 2017) capable of sensing, reacting to, and controlling multiple component activities *in vivo* have required advanced programming paradigms to overcome barriers such as metabolic load (Weinberg et al., 2017), crosstalk (Huh et al., 2013; Kosuri et al., 2013; Trosset and Carbonell, 2013; Brewster et al., 2014), resource sharing (Cardinale et al., 2013; Segall-Shapiro et al., 2014), and gene expression noise (An and Chin, 2009) and thus to grant stability, robustness, and reliability of the engineered systems (Green et al., 2017).

The review is structured in two main sections. The former section summarizes engineering principles that are being applied to devise synthetic genetic circuits. Here, molecular tools exploiting transcriptional, post-transcriptional/translational, and post-translational control mechanisms of gene expression are discussed in separate subsections. The latter section describes specific areas of diagnostic and therapeutic technologies within the precision oncology enterprise where the potential of synthetic biology applications sits at the vanguard.

## From gene switches to computing devices

**Figure 1:**
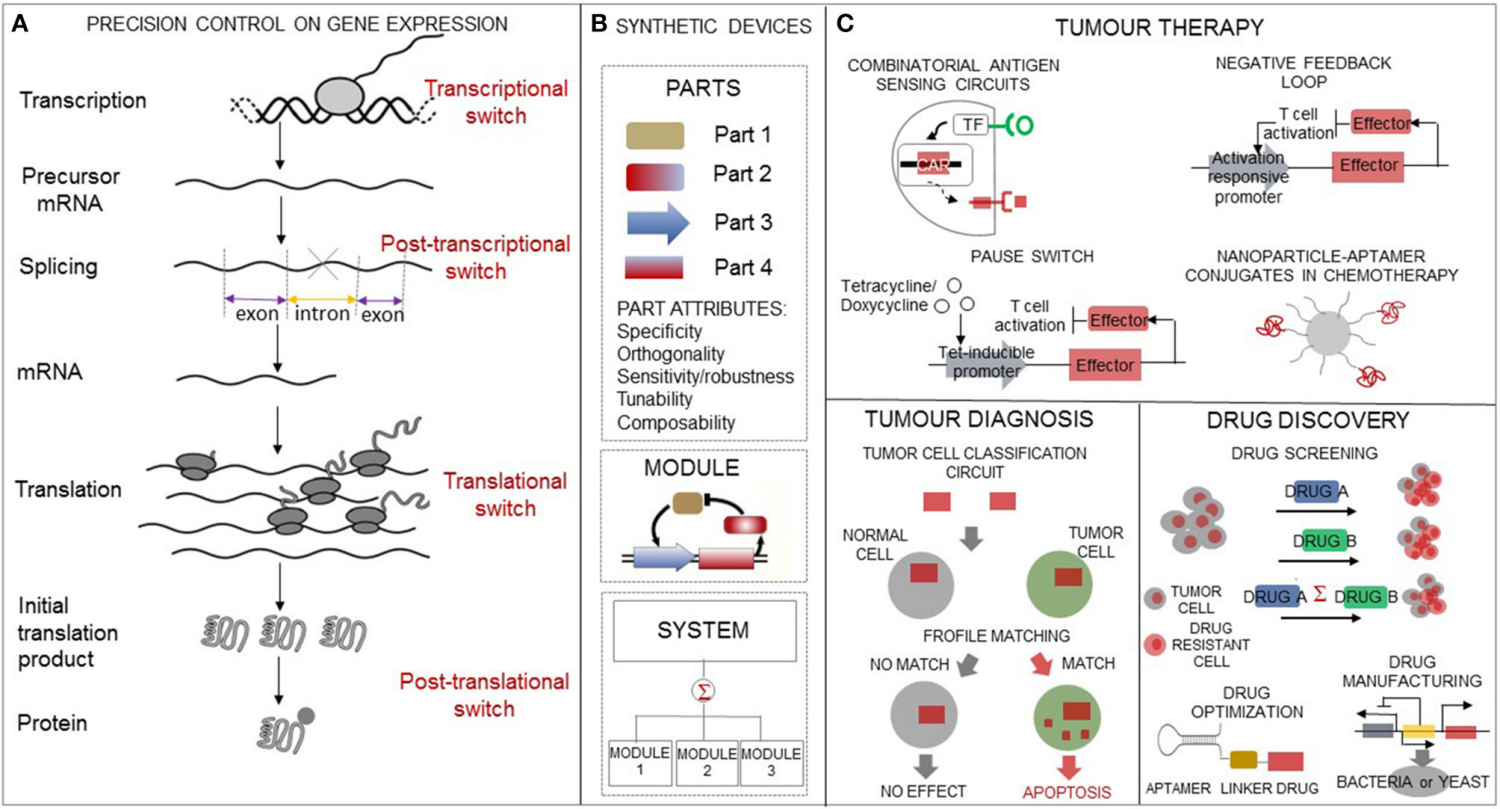
Biological engineering enacts precision tools in oncology. **(A)** The synthetic biology toolbox contains a variety of regulatory switches which allow gene expression control at transcriptional, post-transcriptional, translational, and post-translational levels. **(B)** Abstraction hierarchy used for synthetic circuit design and construction. The hierarchy includes: *parts*, which are endowed with basic biological functions, *devices*, which are any combination of parts that perform a human-defined function, and *systems*, which are any combination of devices. **(C)** Overview of synthetic circuits' applications ranging from drug discovery to tumor diagnosis and to tumor therapy relevant to precision oncology interventions.

Biological engineering has enlarged the molecular tool set available to customize multicomponent constructs with increasingly varied and improved options for controlling gene expression. In particular, a great deal of design effort on synthetic gene switches has allowed to engineer cells with the capacity to sense, process, and switch gene expression state in response to intra- and extracellular signals. Engineering such sensing-actuating constructs involves linking a sensor part that detects the ligand to an actuator part that controls gene expression. The molecular design principles that have been used to customize synthetic gene switches differ according to the gene expression stage at which the switch is applied as well as on the distinctive properties that come with the choice of the switch constitutive parts (Figure 1).

### Tools for transcriptional control

Circuits based on transcriptional control make up the largest number of synthetic circuits and share a common design, where an actuator part enabling positive or negative regulation of transcription is connected with a DNA-binding part that recognizes a promoter DNA sequence. Upon binding of a ligand, a sensor part triggers the activity of this complex through tethering or allosteric mechanisms (Ausländer and Fussenegger, 2013). While native transcription factors have come a long way in synthetic biology applications, it was not until the arrival of programmable transcription factors that it was possible to enhance the engineering capabilities of human-defined transcriptional switches. For example, Zinc-Finger (ZF)-containing factors (Khalil et al., 2012), Transcription Activator-Like Effectors (TALEs; Sanjana et al., 2012; Li et al., 2015), and Clustered Regularly Interspaced Short Palindromic Repeats (CRISPR)-based regulators (Bikard et al., 2013; Qi et al., 2013; Ferry et al., 2017) can be engineered to bind to specific DNA sequences of interest. Each class of TFs comes with advantages and disadvantages and is ideally suited to different applications (Jain, 2013). Major limitations in the application of ZF-containing factors on synthetic circuits are their limited modularity and the lack of specificity of some ZF domains. TALEs are more straightforward to design than ZFs even though they pose challenges to cloning and delivery into host genomes. The CRISPR-based regulators are easier to construct than TALEs which, nonetheless, perform better for the construction of layered circuits (Lebar and Jerala, 2016). The plasmid pT181 antisense-RNA-mediated transcription attenuation platform is well established to control transcription through RNA–RNA interactions (Lucks et al., 2011).

### Tools for post-transcriptional control

Due to its functional diversity, RNA is an advantageous substrate for information sensing, processing, and computation functions. Furthermore, the transient nature of RNA is appealing for applications where safety is a primary concern, since RNA-mediated circuits do not leave a long-term genetic footprint. RNA-based sensing-actuation switches are generally composed of highly folded sensor RNAs (aptamers) that, through conformational changes induced by the binding of small molecules or proteins, regulate the activity of RNA actuators that can operate *in cis* or *in trans*. Switches can sometimes rely on a transmitter part to transduce information between the sensor and actuator (Ogawa and Maeda, 2008). Aptamers have been engineered to respond predominantly to small molecules and nucleic acids (Werstuck and Green, 1998; Win et al., 2009; Shen et al., 2015) with extreme specificity whereas aptamers sensing proteins are far less intensely exploited (Culler et al., 2010).

Actuation can occur through diverse mechanisms including splicing, stability, translation, and mRNA localization. Owing to the known impact of ribonucleases (RNases) on RNA maturation and stability, aptamers have often been combined with RNA substrates for RNase activities (Vazquez-Anderson and Contreras, 2013; Comeau et al., 2016). Many RNA-based devices combine aptamers with catalytic actuators such as self-cleaving ribozymes to achieve flexible regulatory properties to fit application-specific performance requirements (Win and Smolke, 2007; Chen et al., 2010; Ketzer et al., 2012). Aptamers were also used with RNA interference substrates to control target mRNA silencing by regulating Drosha processing of pri-miRNAs (Beisel et al., 2011) or Dicer processing of small hairpin RNAs in response to endogenous signals (Saito et al., 2011). Furthermore, siRNAs and miRNAs have been shown to provide valuable options to implement Boolean logic frameworks (Rinaudo et al., 2007; Xie et al., 2011; Schreiber et al., 2016). A variety of switches have been developed to regulate translation of an open reading frame in response to the binding between the aptamer and small molecule (Stoltenburg et al., 2007; Wroblewska et al., 2015) or protein ligand (Hanson et al., 2003; Win and Smolke, 2007). Translation-control switches mainly affect translation initiation, such as the translational repression/activation switches consisting of the ribosomal protein L7Ae and its box C/D kink-turn binding RNA motif (Saito et al., 2010, 2011). Furthermore, engineered systems can repress and/or activate translation by inducing conformational changes in nascent structured mRNA that modulate the access of the translational machinery to ribosome binding sites (Isaacs et al., 2004; Salis et al., 2009). Enhancement of protein synthesis has been recently achieved by the use of natural and synthetic antisense long non-coding RNAs (Yao et al., 2015) which were named SINEUPs due to the requisite of the inverted SINEB2 sequence to UP-regulate gene-specific translation (Zucchelli et al., 2015). Finally, engineering upstream Open Reading Frames (uORFs), whose regulatory potential is increasingly being appreciated (Re et al., 2016), is predictably an additional exploitable tool for protein manufacturing (Ferreira et al., 2013). Finally, RNA-based devices have been built to enhance gene regulatory activities through co-localization (Lee et al., 1999).

Artificial signal cascades can be constructed by combining multiple regulators, examples of which are inverter modules for synthetic translational switches (Endo et al., 2013). Programming Boolean operators for translational regulation has also been allowed by rationally designed variants of the RNA-IN-RNA-OUT antisense RNA-mediated translation system (Mutalik et al., 2012) as well as by the design of multiple orthogonal ribosome-mRNA pairs (Rackham and Chin, 2005), which were also implemented to synthesize orthogonal transcription-translation networks (An and Chin, 2009).

### Tools for post-translational control

Synthetic switches have been designed that control protein activity by altering protein stability, which for instance is obtained by temporarily tagging proteins with a degradation signal, which guides the protein to the endogenous ubiquitin-proteasome system (Los et al., 2008; Collins et al., 2017). Efforts to engineer phosphorylation-mediated circuitry have been undertaken to rewire and construct MAP kinase circuits (Bashor et al., 2008; Wei et al., 2012; Ryu and Park, 2015). Additionally, the ability of inteins to form and cleave specific peptide bonds is extensively exploited to implement sensors of protein-protein interactions and small molecules, to realize synthetic circuits to deliver CRISPR-Cas9 system components (Truong et al., 2015) and to implement logic gates (Schaerli et al., 2014). Further efforts are ongoing to engineer and characterize synthetic compartmentalization approaches providing veritable solutions to implement modularity in synthetic devices (Chen and Silver, 2012).

## Synthetic circuit-based tools for precision medicine in oncology

We outline synthetic biology applications which are expanding existing options in cancer diagnosis, cancer therapeutics, and for pharmaceutical compound screening (Figure 1).

### Tumor diagnosis

Precise cell state discrimination is essential for *in vivo* targeting of cancer cells. Medical diagnosis based on individual elements is unavoidably thwarted by lack of specificity and sensitivity. Therefore, diagnostic algorithms are being formalized using combinatorial Boolean logic to perform integrated detection and analysis of multiple signals in living cells (Rubens et al., 2016; Schreiber et al., 2016). Expression profiles are widely used to drive decision-making circuits such as the multi-input RNAi-based logic circuit identifying specific cancer cells (Xie et al., 2011). The cancer classifier circuit implemented in this study selectively triggers either a fluorescent reporter or apoptosis in HeLa cells. More precisely, this circuit integrates sensory information from six endogenous microRNAs to determine whether a cell matches a pathological reference pattern characteristic of the HeLa cervical cancer cell line and, if so, produces an apoptotic response. Early efforts to develop bio-based computing capabilities such as counting (Friedland et al., 2009) and memory storage (Siuti et al., 2013) lead to the notion that bacterial cells could become diagnostic indicators for recording exposure events (Cronin et al., 2012). In one of such studies, probiotic bacteria were transformed with a dual-stabilized, high-expression lacZ vector, and an integrated luxCDABE cassette endowing luminescent visualization in order to target, visualize, and diagnose liver metastasis (Danino et al., 2015). A recent study brought whole-cell biosensor closer to clinical requirements by configuring digital amplifying genetic switches, based on transistor-like three terminal devices (Bonnet et al., 2013), to actuate logic gates in bacterial chasses (Courbet et al., 2015). Here, digital amplifying switches are used in Boolean logic gates to perform complex signal processing tasks such as multiplexed detection of clinically relevant markers, signal digitization, and amplification along with storage of the medically informed outcome in a stable DNA register for *a posteriori* interrogation. Standardized devices for cancer diagnosis require a great deal of fine-tuning efforts to make combinatorial logic gates to perform as intended. Therefore, progressively advanced studies are being reported, opening interesting avenues to the automation of combinatorial circuit engineering (Ausländer et al., 2012; Nielsen et al., 2016; Weinberg et al., 2017). Even so, there are cumbersome problems that still need to be dealt with. Despite the breadth and depth described above, it is difficult to control the trade-off between specificity and sensitivity achieved by expression-based cell classifier designs, the changes in constructs performance dependent on genetic context, space and time as well as the possible toxicity induced by regulators overexpression. Balancing these problems must be addressed in order to allow synthetic gene constructs to become part of a personalized cancer therapy toolbox.

### Tumor therapy

Synthetic biology is primed to provide the conceptual framework and genetic tools necessary to enhance cell- (Fischbach et al., 2013) and gene- (Costales et al., 2017) based therapeutics.

#### Cell-based therapeutics

Immunotherapy has shown great promise for eradicating tumor in clinical trials. Much of the current success derives from therapies based on engineering T cell receptors (TCRs) and chimeric antigen receptors (Wilkie et al., 2012; Kloss et al., 2013; Duong et al., 2015; CARs), that consist of a cancer antigen-specific single-chain variable fragment (scFv) fused to a T cell signaling domain that triggers activation and proliferation. Nowadays, synthetic sensors, switches, and circuits are primed to improve T cell therapy efficacy and meet safety concerns (e.g., discriminative capacity between tumors and vital organs and potential adverse side effects) by providing inducible control over the specificity, localization, duration, and extent of T cell activities.

##### Receptor systems

One of the most important challenges is represented by cell specificity. A powerful way to enhance on-target activity of therapeutic T cells is to engineer combinatorial receptor systems such as dual receptor AND-gate T cells (Roybal et al., 2016). In the antibody-coupled T cell receptor (ACTR) system, the scFv is replaced with the extracellular portion of CD16, a receptor that binds to the constant fragment of antibodies so that any relevant cancer-specific antibody can, in principle, be administered upon antigen binding (Kudo et al., 2014). Another major concern is the potential risk of unpredictable therapeutic effect. To enhance controllability, the recent GoCAR-T system incorporates a switch that activates CAR T cells when it is triggered not only by the target antigen expressed on the surface of the cancer cells but also by controlled administration of the drug rimiducid (Foster et al., 2017).

##### Control switches and circuits

T cell therapies could meet safety concerns if it were possible to eliminate quickly the engineered cells upon adverse side effects. Drug-inducible kill switches are an interesting development to achieve this goal. A recent example employs an inducible caspase 9 in conjunction with a CD20-specific CAR to test *in vivo* its potential to remove CAR-bearing T cells (Budde et al., 2013). Another study proposed to fuse caspase 9 to a modified FK-binding protein in order to allow conditional dimerization. This construct was proven to lead to cell death when exposed to a dimerizing small molecule (Di Stasi et al., 2011).

The design of negative feedback loops and inducible pause switches is proving a useful alternative to T cell elimination by modulating the immune response amplitude and timing. These circuits exploit the ability of bacterial virulence effector proteins to evade the immune response. The former type creates a negative feedback loop by expressing these proteins under the control of a T cell activation responsive promoter (Wei et al., 2012). The latter type of circuits pauses T cell activation by expressing bacterial virulence proteins under the control of a tetracycline inducible promoter. Indeed, adding the drug leads to the expression of the effector proteins, which in turn stop cell activation until the drug is removed (Wei et al., 2012).

Finally, a potent tool to regulate the therapy safety and efficacy is provided by growth switches (Chen et al., 2010). Here, a ribozyme drives self-cleavage of the cytokine transcript and leads to cytokine expression shut off; adding a proper drug prevents self-cleavage so that cytokines are expressed and lead to T cell proliferation.

#### Gene-based therapeutics

Gene circuit engineering has greatly improved our ability to programme genes involved in tumor origin and progress. For instance, some high-affinity RNA aptamers against PPAR-δ, a lipid-sensing nuclear receptor involved in cancer (Kwak et al., 2009), β-catenin (Lee et al., 2006), and nucleolin (Soundararajan et al., 2008), could lead to reduction of tumor-forming potential. Furthermore, a computational workflow, that selects RNA motif-small molecule binding interactions by library-vs.-library screening (2DCS) and then mines them against RNA folds in the transcriptome, allowed to identify a small molecule inhibitor of an oncogenic non-coding RNA (Velagapudi et al., 2017). SiRNAs can also specifically bind to target genes but their application can be limited by the absence of effective vehicles. For this purpose, several studies have proposed the use of aptamers in siRNA expressing constructs as vehicles (Tai and Gao, 2016).

### Drug delivery

Today nanobiotechnology provides extremely versatile options to address the localized delivery of genetically encoded tools such as virus-based vectors modified to carry engineered payloads (Ryan et al., 2004; Li et al., 2005), oncolytic viruses exploiting dual promoter logics (Nissim and Bar-Ziv, 2010), and nanoparticle-aptamer bioconjugates (Farokhzad et al., 2006). Recently, (Douglas et al., 2012) described a shape-switching device for targeted transport of signaling molecules. The robotic DNA device consists of a barrel provided with DNA aptamer-based locks that open in response to the binding of cell type-specific antigen keys. Vibrant developments greatly enhance and wide the range of applicable dynamic DNA and RNA-based nanoparticles (Afonin et al., 2013; Edwardson et al., 2016) besides opening newer avenue to conjugate inter-dependent nanoparticles (Halman et al., 2017). Polymer materials responsive to external signals (Stuart et al., 2010) such as nanogels conjugated to ligands recognized by cell specific receptors (Oishi et al., 2007), virus-mimetic nanogels (Lee et al., 2008), and hydrogels based on ligand-responsive DNA–protein interactions (Christen et al., 2011) demonstrate the essential progress in the area. In the future, nanorobots could be routed toward the tumor by exploiting the tumor-homing ability of self-propelled bacteria, similar to a recent study (Katuri et al., 2017). Biological vesicles derived from mammalian cells have also attracted much attention for *in vivo* delivery (Yoo et al., 2011). In particular, exosomes (Wang et al., 2016) have been engineered to deliver chemotherapeutics to tumor tissue in mouse models for cancer (Tian et al., 2014).

### Drug discovery

Synthetic biology is helping to address previously unfeasible challenges the field of drug discovery. Progress on design of synthetic genetic circuits (Carbonell et al., 2014; Trosset and Carbonell, 2015) has opened the possibility of their use not only for production of drugs (Breitling and Takano, 2015) but also for the development of platforms for identification and validation of drug targets (Firman et al., 2012; Kasap et al., 2014) as well as for phenotypic cell-based screening approaches (Duportet et al., 2014) such as the screening for anti-cancer drugs presented in Gonzalez-Nicolini et al. (2004), that discriminates between proliferation competent and mitotically inert cells and eliminates preferentially neoplastic ones. With this purpose, (Gonzalez-Nicolini et al., 2004) engineered a transgenic CHO-K1-derived cell line to enable G1-specific growth arrest conditioned on the tetracycline responsive overexpression of the human cyclin-dependent kinase inhibitor p27. Another study applied a one-bead-two-compound (OB2C) cell-based screening approach for the discovery of synthetic molecules that can interact with cellular receptors as well as enhance or inhibit downstream cell signaling (Kumaresan et al., 2011). The primary innovation of this system is represented by the usage of beads provided with two chemical molecules on the surface and a chemical tag to probe cellular responses. When cells are incubated with the OB2C library, a cell adhesion ligand captures live cells on each bead in the library. The bound cells can interface with the tethered OB2C library compounds and then be probed for specific cellular signaling pathways such as leukemic cell death responses (Kumaresan et al., 2011). Largely because of similar progresses in conceptual design and technologies, synthetic biology is being employed as a powerful way to identify drug mechanisms of action and to accelerate the development of drug combination-based approaches (Chandrasekaran et al., 2016).

## Conclusions

In this review, we focus on advances in biological engineering which stimulated the development of innovative approaches for precision intervention in oncology. The growing contribution of synthetic biology to drug discovery as well as the widening availability of synthetic circuits, which are already being used in different human compatible cell types and animal models for safe operation of gene- and cell-based therapies, demonstrate the potential of future approaches integrating systems and synthetic biology tools to precisely match therapies to individual cancer patients.

## Author contributions

The author confirms being the sole contributor of this work and approved it for publication.

### Conflict of interest statement

The author declares that the research was conducted in the absence of any commercial or financial relationships that could be construed as a potential conflict of interest.
